# Dosing Pattern and Early Cumulative Dose of Liposomal Irinotecan in Metastatic Pancreatic Cancer: A Real-World Multicenter Study

**DOI:** 10.3389/fonc.2022.800842

**Published:** 2022-06-22

**Authors:** Yung-Yeh Su, Nai-Jung Chiang, Chung-Pin Li, Chia-Jui Yen, Shih-Hung Yang, Wen-Chi Chou, Jen-Shi Chen, Tai-Jan Chiu, Yen-Yang Chen, Shih-Chang Chuang, Li-Yuan Bai, Chang-Fang Chiu, Cheng-Ming Peng, De-Chuan Chan, Sz-Chi Chiu, Yi-Hsin Yang, Yan-Shen Shan, Li‐Tzong Chen

**Affiliations:** ^1^ National Institute of Cancer Research, National Health Research Institutes, Tainan, Taiwan; ^2^ Department of Oncology, National Cheng Kung University Hospital, College of Medicine, National Cheng Kung University, Tainan, Taiwan; ^3^ Institute of Clinical Medicine, College of Medicine, National Cheng Kung University, Tainan, Taiwan; ^4^ Department of Oncology, Taipei Veterans General Hospital, Taipei, Taiwan; ^5^ School of Medicine, College of Medicine, National Yang Ming Chiao Tung University, Taipei, Taiwan; ^6^ Division of Clinical Skills Training, Department of Medical Education, Taipei Veterans General Hospital, Taipei, Taiwan; ^7^ Division of Gastroenterology and Hepatology, Department of Medicine, Taipei Veterans General Hospital, Taipei, Taiwan; ^8^ Department of Oncology, National Taiwan University Hospital, Taipei, Taiwan; ^9^ Division of Hematology-Oncology, Department of Internal Medicine, Linkou Chang Gung Memorial Hospital and Chang Gung University, Taoyuan, Taiwan; ^10^ Division of Hematology-Oncology, Department of Internal Medicine, Kaohsiung Chang Gung Memorial Hospital and Chang Gung University, Kaohsiung, Taiwan; ^11^ Division of General and Digestive Surgery, Department of Surgery, Kaohsiung Medical University Hospital, Kaohsiung, Taiwan; ^12^ Department of Surgery, Faculty of Medicine, Kaohsiung Medical University, Kaohsiung, Taiwan; ^13^ Division of Hematology and Oncology, Department of Internal Medicine, China Medical University Hospital, and China Medical University, Taichung, Taiwan; ^14^ College of Medicine, School of Medicine, China Medical University, Taichung, Taiwan; ^15^ Cancer Center, China Medical University Hospital, Taichung, Taiwan; ^16^ Department of Surgery, Chung Shan Medical University Hospital and Chung Shan Medical University, Taichung, Taiwan; ^17^ Division of General Surgery, Department of Surgery, Tri-Service General Hospital, National Defense Medical Center, Taipei, Taiwan; ^18^ Department of Sales and Marketing, PharmaEngine, Inc., Taipei, Taiwan; ^19^ Division of General Surgery, Department of Surgery, National Cheng Kung University Hospital, College of Medicine, National Cheng Kung University, Tainan, Taiwan; ^20^ Department of Internal Medicine, Kaohsiung Medical University Hospital, College of Medicine, Kaohsiung Medical University, Kaohsiung, Taiwan; ^21^ Center for Cancer Research, Kaohsiung Medical University, Kaohsiung, Taiwan

**Keywords:** pancreatic cancer, nal-IRI, dose intensity, dose escalation strategy, real world

## Abstract

**Introduction:**

This multicenter, real-world cohort study aimed to evaluate the effectiveness of early cumulative dose administration and dosing pattern of liposomal irinotecan plus fluorouracil/leucovorin (nal-IRI+5-FU/LV) in patients with gemcitabine-refractory metastatic pancreatic ductal adenocarcinoma (mPDAC).

**Material and Methods:**

The electronic medical records of mPDAC patients treated with nal-IRI+5-FU/LV in nine participating centers were manually reviewed. To accommodate to the NAPOLI-1 study population, only patients with an Eastern Cooperative Oncology Group Performance Score of 0–1 were included. The survival impact of the relative 6-week cumulative dose and dosing pattern (standard vs. reduced starting dose, with and without further dose modification) were investigated.

**Results:**

Of the 473 included patients, their median overall survival (mOS) was 6.8 [95% CI, 6.2–7.7] months. The mOS of patients who received a relative 6-week cumulative dose of >80%, 60%–80%, and <60% were 7.9, 8.2, and 4.3 months, respectively (p<0.0001). Their survival impact remained significant after covariate adjustment using Cox regression. The mOS was 8.0–8.2 months in patients with a standard starting dose with and without early dose modification, and 9.3 and 6.7 months in those who had a reduced starting dose with and without escalation in the subsequent treatment, respectively. The incidence of grade 3–4 neutropenia and diarrhea was 23.3% and 2.7%, respectively.

**Conclusion:**

Our results support the use of nal-IRI+5-FU/LV in gemcitabine-refractory mPDAC and suggest that a lower starting dose followed by a re-escalation strategy could achieve clinical outcomes comparable to those with standard starting doses in real-world practice.

## Introduction

Pancreatic ductal adenocarcinoma (PDAC) is the third leading cause of cancer mortality in the United States and the fourth in Japan and the European Union in 2020. It is predicted to be the second leading cause by 2030 in the United States and Germany (1–5). Owing to the delay in diagnosis and lack of effective therapeutic regimens, PDAC remains one of the deadliest cancers over the years. In the pivotal randomized phase 3 NAPOLI-1 study, liposomal irinotecan plus 5-fluorouracil/leucovorin (nal-IRI+5-FU/LV) demonstrated significant survival benefits in patients with gemcitabine-refractory metastatic PDAC (mPDAC) (6). Currently, nal-IRI+5-FU/LV is the only approved regimen for pancreatic cancer after the failure of gemcitabine-based treatment. Although randomized controlled trials remain the gold standard for drug approval, it has frequently been questioned whether the results of clinical trials can be reproducible in daily practice.

The use of medical information from routine healthcare settings has provided good support for filling the gap between clinical trials and real-life practice. Both the European Medicines Agency and the US Food and Drug Administration have adopted real-world evidence in the regulatory process (7, 8). The results of 11 real-world data (RWD) on the effectiveness of nal-IRI+5-FU/LV in pancreatic cancer from different regions and ethnicities are summarized in **Supplementary Table 1** (9–19). However, due to the heterogeneity of study populations, that is, 0%–27.3% of patients with Eastern Cooperative Oncology Group performance score (ECOG PS) ≥2 and 0%–69.6% with a reduced starting dose, these clinical outcomes may not be directly compared (20).

The impact of early dose modification on the clinical outcomes of patients receiving nal-IRI+5-FU/LV remains unclear. For example, in the pre-specified analysis of the NAPOLI-1 study, the median overall survival (mOS) of per-protocol (PP) patients who received ≥80% of planned treatment within the first 6 weeks was 8.9 (95% confidence interval [CI], 6.4–10.5) months, as compared to the 4.4 (95% CI, 3.3–5.3) months of the non-PP cohort (20). On the other hand, the mOS of patients who had at least two dose treatments in the NAPOLI-1 study with and without early dose modification was 8.4 (95% CI, 5.26–11.04) and 6.7 (95% CI, 4.70–8.87) months, respectively (21). Therefore, we address the impact of a 6-week cumulative dose administration and different dosing patterns on the survival of patients enrolled in a large real-world multicenter cohort study.

## Materials and Methods

### Patients

This was a multicenter retrospective observational study; the inclusion criteria were as follows: 1) the receipt of nal-IRI+5-FU/LV after reimbursement in August 2018, 2) prior exposure to gemcitabine, 3) the presence of metastasis upon treatment with nal-IRI+5-FU/LV. The exclusion criteria were as follows: 1) nal-IRI+5-FU/LV in combination with other anti-cancer agents, 2) the substitution of 5-FU with S1, and 3) no metastasis upon the treatment of nal-IRI+5-FU/LV. This retrospective study was approved by the Institutional Review Board (IRB) of all participating institutes following the Declaration of Helsinki; informed consent was waived due to its retrospective nature. The IRB approval number of the institutes was as follows: Chang Gung Memorial Hospital 202100783B0; China Medical University Hospital CMUH109-REC2-176; Chung Shan Medical University Hospital CS2-21095; National Cheng Kung University Hospital A-ER-109-477; National Taiwan University Hospital 201911042RINC; Kaohsiung Medical University Hospital KMUHIRB-E(I)-20210150; Taipei Veterans General Hospital 2021-08-001AC; and Tri-Service General Hospital B202105057.

### Assessment

Patient demographics, treatment outcomes, and adverse events were manually reviewed using electronic medical records. Upon the discretion of the physician, a radiographic evaluation of the tumor response was performed every 8–12 weeks by using the Response Evaluation Criteria for Solid Tumors version 1.1. Adverse events were evaluated and recorded according to the Common Terminology Criteria for Adverse Events version 4.0.3 (20).

### Statistical Analysis

The descriptive statistics of patient demographics are presented as medians or percentages, as appropriate. The normality of the data distribution was checked using the Kolmogorov–Smirnov test. Fisher’s exact test was used to compare the differences in proportions between the groups.

The median duration of follow-up was estimated using the reverse Kaplan–Meier method. Progression-free survival (PFS) was defined as the time between the start date of nal-IRI+5-FU/LV and the date of disease progression, death, intolerance, loss of follow-up, or end of study, whichever occurred first. OS was calculated from the start of nal-IRI+5-FU/LV to death, loss to follow-up, or end of the study. PFS data was censored for intolerance, the loss of follow-up, or end of the study. OS data were censored for loss to follow-up or the end of the study. PFS and OS were estimated using the Kaplan–Meier method. Survival differences between groups were compared using the log‐rank test.

To investigate the effect of the cumulative dose, Cox regression analysis was used to estimate the hazard ratio (HR) and 95% CI. All variables with *p* < 0.05 were statistically significant. All statistical analyses were performed using R version 4.0.5 (R Core Team, Vienna, Austria) and SAS version 9.4 (SAS Institute Inc., Cary, NC, United States).

## Results

### Baseline Characteristics

**Figure 1:**
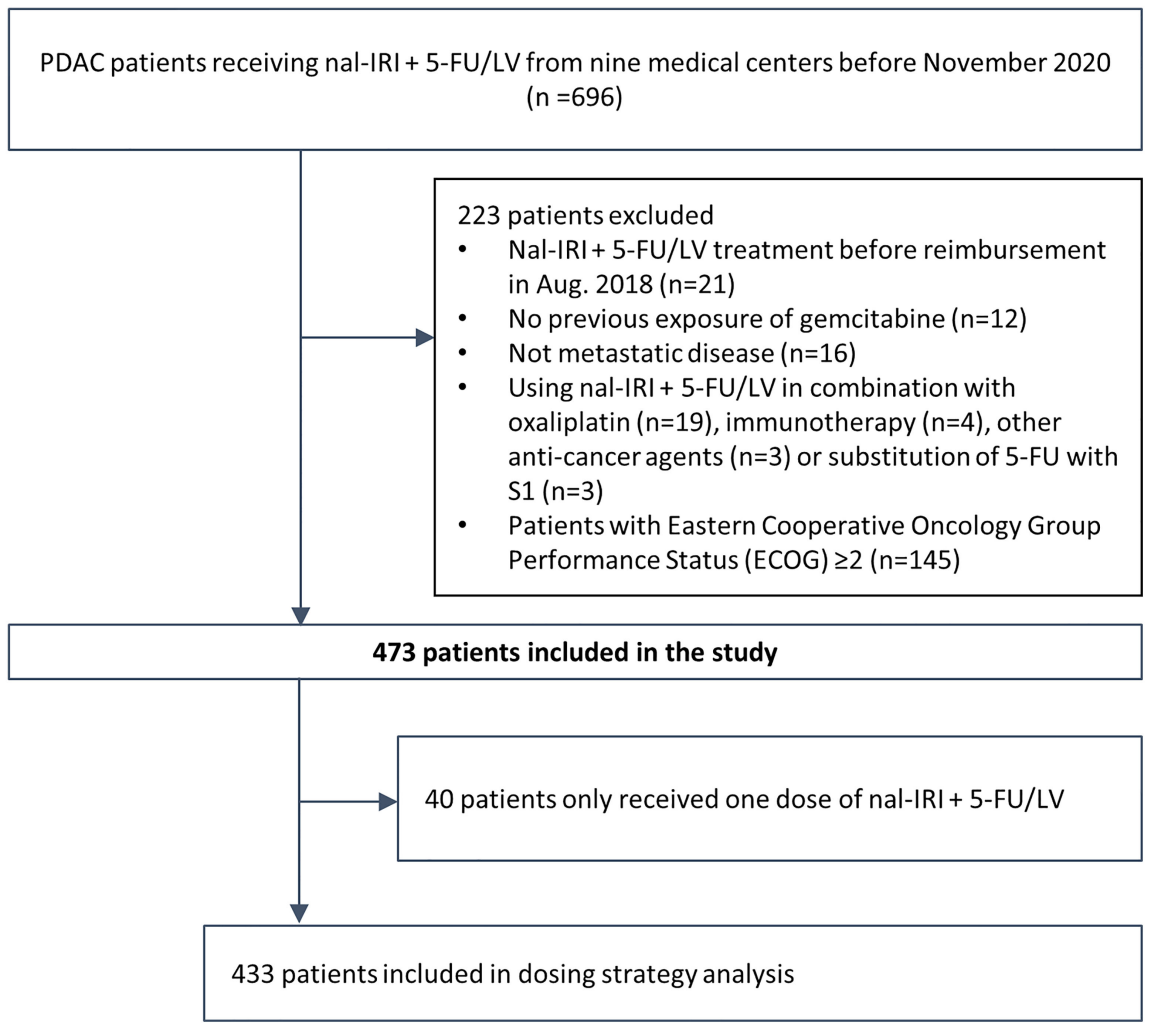
Flowchart of study enrollment.

The electronic medical records (EMRs) of 696 patients treated with nal-IRI+5-FU/LV from nine medical centers in Taiwan before November 2020 were retrospectively reviewed. To achieve better cohort homogeneity, only patients treated with nal-IRI+5-FU/LV after reimbursement in August 2018 were included. After the exclusion of 78 patients who did not fulfill the inclusion criteria, 618 patients with gemcitabine-refractory metastatic pancreatic cancer treated with nal-IRI+5-FU were included. For better comparison with the NAPOLI-1 study, in which only patients with a Karnofsky PS score ≥70 (equivalent to ECOG PS 0 or 1) were eligible, 145 patients with ECOG PS ≥2, who had significantly inferior OS (mOS 2.6 [95% CI, 2.2–3.1] vs. 6.8 [95% CI, 6.2–7.7] months of patients with ECOG PS 0–1, were excluded from the study (**Supplementary Figure S1**) (22). Finally, a total of 473 patients were included in the study (**Figure 1**). All baseline characteristics were comparable between the current and the NAPOLI-1 study; however, our patients had more stage IV disease at diagnosis and with prior exposure to fluorouracil-containing treatment than those in the NAPOLI-1 study (67.4% vs. 52.1% and 77.4% vs. 42.7%, respectively) (**Table 1**) (6, 23).

**Table 1:** Baseline characteristics.

	NAPOLI-1 Nal-IRI+5-FU/LV (N = 117)	Real-world cohort				
		Overall(N = 473)	6-week cumulative dose >80% (N = 175)	6-week cumulative dose 60%–80% (N = 134)	6-week cumulative dose <60% (N = 164)	P-value §
**Gender**						0.238
Female	48 (41%)	198 (41.9%)	81 (46.3%)	56 (41.8%)	61 (37.2%)	
Male	69 (59%)	275 (58.1%)	94 (53.7%)	78 (58.2%)	103 (62.8%)	
**Age**, year, median (range)	63 (41–81)	63 (27–86)	62 (27–80)	64 (37–86)	63 (34–82)	0.506
**Disease stage at diagnosis**						0.005
Stage I–III	56 (47.9%)	154 (32.6%)	56 (32.0%)	31 (23.1%)	67 (40.9%)	
Stage IV	61 (52.1%)	319 (67.4%)	119 (68.0%)	103 (76.9%)	97 (59.1%)	
**Primary tumor location^†^**						0.374
Head	70 (59.8%)	249 (52.6%)	87 (49.7%)	75 (56.0%)	87 (53.0%)	
Body	12 (10.3%)	108 (22.8%)	41 (23.4%)	32 (23.9%)	35 (21.3%)	
Tail	14 (12.0%)	93 (19.7%)	42 (24.0%)	19 (14.2%)	32 (19.5%)	
Multi-locations including head	6 (5.1%)	20 (4.2%)	5 (2.9%)	6 (4.5%)	9 (5.5%)	
Multi-locations excluding head	9 (7.7%)	3 (0.6%)	0 (0%)	2 (1.5%)	1 (0.6%)	
**ECOG**						-
0-1	114 (97.4%)	249 (52.6%)	175 (100%)	134 (100%)	164 (100%)	
≥2	3 (2.6%)	-	-	-	-	
**Baseline albumin**						<0.001
≥4.0 g/dl	53 (45.3%)	114 (24.1%)	35 (20.0%)	38 (28.4%)	41 (25.0%)	
<4.0 g/dl	64 (54.7%)	179 (37.8%)	55 (31.4%)	38 (28.4%)	86 (52.4%)	
Not recorded	-	180 (38.1%)	85 (48.6%)	58 (43.3%)	37 (22.6%)	
**Number of metastatic sites**						0.632
1	19 (16.2%)	254 (53.7%)	87 (49.7%)	77 (57.5%)	90 (54.9%)	
2	49 (41.9%)	141 (29.8%)	54 (30.9%)	37 (27.6%)	50 (30.5%)	
3	22 (18.8%)	65 (13.7%)	27 (15.4%)	16 (11.9%)	22 (13.4%)	
≥4	7 (6.0%)	13 (2.7%)	7 (4.0%)	4 (3.0%)	2 (1.2%)	
**Sites of metastases**						
Liver	75 (64.1%)	319 (67.4%)	116 (66.3%)	79 (59.0%)	124 (75.6%)	0.009
Lung	36 (30.8%)	122 (25.8%)	48 (27.4%)	38 (28.4%)	36 (22.0%)	0.373
Peritoneum	28 (23.9%)	130 (27.5%)	51 (29.1%)	41 (30.6%)	38 (23.2%)	0.298
Distant node	32 (27.4%)	135 (28.5%)	58 (33.1%)	35 (26.1%)	42 (25.6%)	0.235
**CA 19-9**						0.029
<40 U/ml	22 (18.8%)	69 (14.6%)	25 (14.3%)	16 (11.9%)	28 (17.1%)	
≥40 U/ml	92 (78.6%)	346 (73.2%)	120 (68.6%)	100 (74.6%)	126 (76.8%)	
Not recorded	3 (2.6%)	58 (12.3%)	30 (17.1%)	18 (13.4%)	10 (6.1%)	
**Previous anticancer therapy**						
mFOLFIRINOX	NA	38 (8.0%)	12 (6.9%)	5 (3.7%)	21 (12.8%)	0.013
Gem + nab-P	NA	144 (30.4%)	49 (28.0%)	33 (24.6%)	62 (37.8%)	0.033
SLOG	NA	97 (20.5%)	49 (28.0%)	28 (20.9%)	20 (12.2%)	0.002
Gem + S1	NA	132 (27.9%)	53 (30.3%)	40 (29.9%)	39 (23.8%)	0.344
Gemcitabine-containing	117 (100%)	473 (100%)	175 (100%)	134 (100%)	164 (100%)	-
Fluorouracil-containing	50 (42.7%)	366 (77.4%)	141 (80.6%)	106 (79.1%)	119 (72.6%)	0.181
S1-containing	NA	317 (67.0%)	129 (73.7%)	93 (69.4%)	95 (57.9%)	0.007
Irinotecan-containing	12 (10.3%)	64 (13.5%)	17 (9.7%)	14 (10.4%)	33 (20.1%)	0.009
Platinum-containing	38 (32.5%)	218 (46.1%)	85 (48.6%)	66 (49.3%)	67 (40.9%)	0.249
**Prior lines of advanced diseases** ‡						0.014
0	62 (53%)	295 (62.4%)	120 (68.6%)	83 (61.9%)	92 (56.1%)	
1	15 (13%)	7 (1.5%)	0 (0%)	5 (3.7%)	2 (1.2%)	
≥2	40 (34%)	171 (36.2%)	55 (31.4%)	46 (34.3%)	70 (42.7%)	
**Prior surgery**						0.036
No surgery	NA	281 (59.4%)	111 (63.4%)	79 (59.0%)	91 (55.5%)	
Whipple operation	30 (26%)	77 (16.3%)	27 (15.4%)	29 (21.6%)	21 (12.8%)	
Other surgical procedure	NA	115 (24.3%)	37 (21.1%)	26 (19.4%)	52 (31.7%)	
**Time since last previous therapy**						0.366
Median (IQR), months	1.4 (0.9-2.1)	0.7 (0.5–1.2)	0.7 (0.5–1.0)	0.7 (0.5–1.2)	0.7 (0.5–1.2)	
Not recorded	-	65 (13.7%)	19 (10.9%)	30 (22.4%)	16 (9.8%)	

IQR, interquartile range; NA, not available.

^†^In the NAPOLI-1 study, 2.7% of patients are classified as having an unknown location, which is not shown here.

^‡^The definition is slightly different. Systemic therapy used for locally advanced disease is counted in the present real-world study but not in the NAPOLI-1 study. §P-value for subgroups

### Relative 6-Week Cumulative Dose Significantly Impacts Outcome

**Figure 2:**
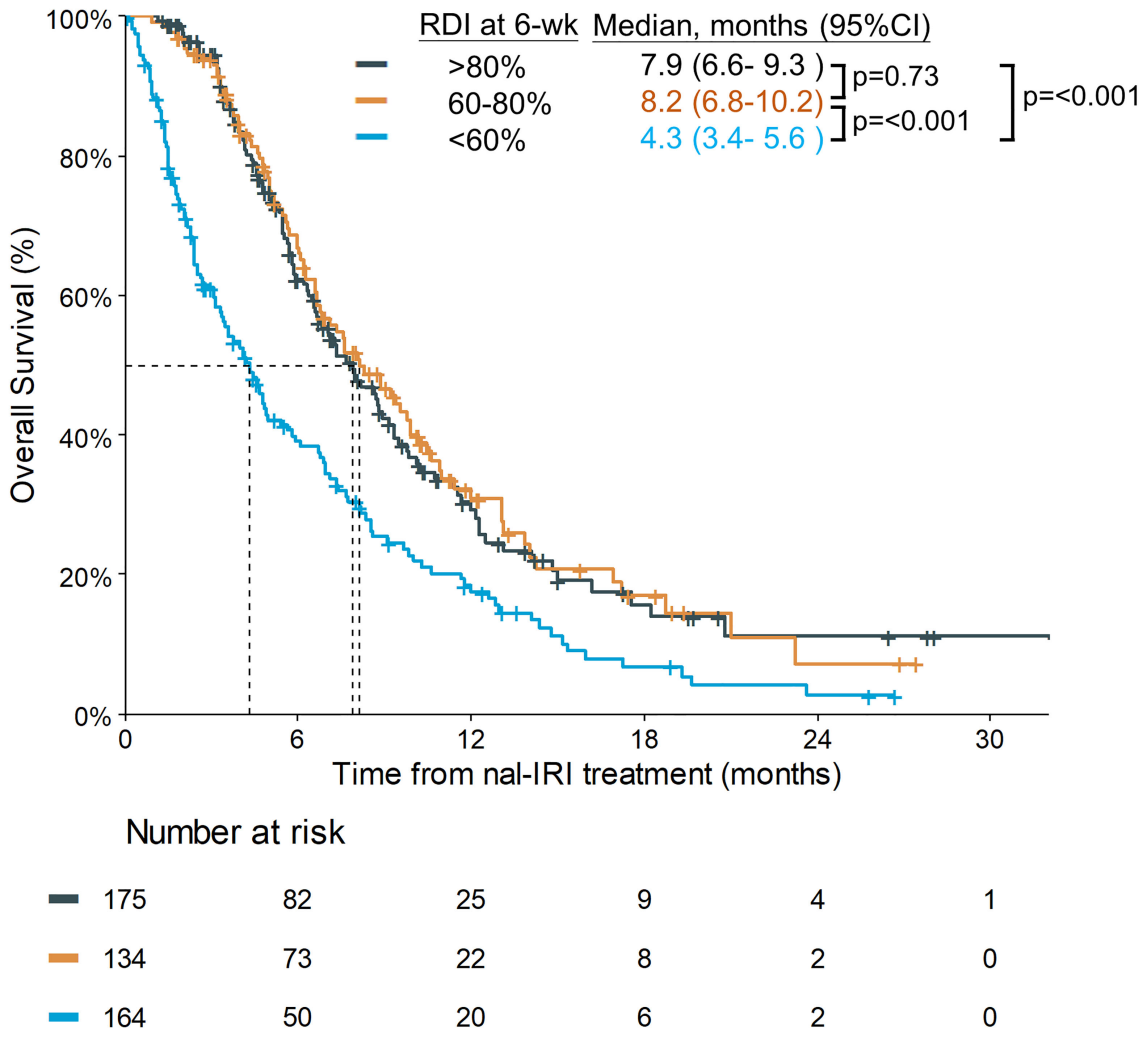
Overall survival (OS) of different cumulative dose groups. RDI, relative dose intensity.

The planned 6-week cumulative dose of nal-IRI was 240 mg/m^2^, equivalent to a 210 mg/m^2^ irinotecan free base. The PP analysis of the NAPOLI-1 study defined patients who received ≥80% of the planned nal-IRI dose during the first 6 weeks as the PP population. Therefore, we set a relative 6-week cumulative dose of 80% (192 mg/m^2^) as the first cutoff point and further divided the patients by the second cutoff points of 60% (144 mg/m^2^). As the data cutoff date was December 31, 2020, the median duration of the follow-up was 13.1 months [interquartile range (IQR) 7.0–20.6 months]. The mOS was significantly different in patients who received a relative 6-week cumulative dose >80%, 60%–80%, and <60%, 7.9 (95%CI, 6.6–9.3) vs. 8.2 (95%CI, 6.8–10.2) vs. 4.3 (95%CI, 3.4–5.6) months (p<0.0001) (**Figure 2**). The objective response rate (ORR) for the entire cohort was 9.1%. The median number of treatment cycles was 5; the median duration of treatment was 10.3 weeks, which was numerically better than the NAPOLI-1 study. The median 6-week cumulative dose of nal-IRI (salt based) was 170 mg/m^2^, as compared to 167.5 mg/m^2^ in the NAPOLI-1 study (**Table 2**).

**Table 2:** Treatment response and dose.

	NAPOLI-1 Nal-IRI+5-FU/LV (N = 117)	Real-world cohort				
		Overall (N = 473)	Relative 6-week cumulative dose >80% (N = 175)	Relative 6-week cumulative dose 60-80% (N = 134)	Relative 6-week cumulative dose <60% (N = 164)	P-value for subgroups
**Best overall response**						<0.001
Complete response	0	1 (0.2%)	1 (0.6%)	0 (0%)	0 (0%)	
Partial response	19 (16.2%)	42 (8.9%)	14 (8.0%)	17 (12.7%)	11 (6.7%)	
Stable disease	39 (33.3%)	139 (29.4%)	68 (38.9%)	30 (22.4%)	41 (25.0%)	
Progression disease	34 (29.1%)	231 (48.8%)	76 (43.4%)	77 (57.5%)	78 (47.6%)	
Not evaluable	22 (18.8%)	60 (12.7%)	16 (9.1%)	10 (7.5%)	34 (20.7%)	
**Number of cycles, median (IQR)**	3 (2–9)	5 (3–9)	6 (4–11)	6 (4–10)	3 (2–6)	<0.001
**Duration of treatment,** **weeks, median (IQR)**	8.7 (5.4–22.0)	10.3 (5.7–22.0)	13.0 (8.2–26.4)	12.8 (7.0–27.1)	5.9 (1.6–13.4)	<0.001
**6-week cumulative dose,** **median (IQR), mg/m^2^**	167.5 (IQR not disclosed)	170 (122–210)	220 (205–236)	166 (158–183)	102 (79.5–123)	<0.001

IQR, interquartile range.

### Covariate Adjustment

As baseline characteristics were not balanced among the three cumulative dose groups, univariate Cox regression analysis was performed to identify possible covariate effects (**Table 3**). A multivariable Cox regression for OS was constructed using factors that were significantly predictive of OS by univariate analysis (p < 0.05) or that were considered clinically important; stage IV upon diagnosis, hypoalbuminemia (<4.0 g/dl), and CA 19-9 ≥ median (921 U/ml) were the most important prognostic factors in the multivariable analysis (**Table 3**). The prognostic impact of the 6-week cumulative dose remained significant after covariate adjustment (>80% vs. <60%, HR 2.12, 95% CI: 1.60–2.81, p<0.001).

**Table 3:** Univariate and multivariable Cox regression of overall survival.

	Univariate		Multivariable	
	HR (95% CI)	p-value	HR (95% CI)	p-value
**Gender (female vs. male)**	1.38 (1.10 - 1.73)	0.006	1.28 (1.00 - 1.62)	0.05
**Age**	1.00 (0.99 - 1.02)	0.5	1.01 (0.99 - 1.02)	0.41
**Disease stage at diagnosis (Stage I-III vs. Stage IV)**	1.51 (1.19 - 1.92)	<0.001	1.52 (1.17 - 1.96)	0.001
**Primary tumor location (Head vs. Others)**	1.41 (1.13 - 1.76)	0.002	1.09 (0.85 - 1.40)	0.49
**Baseline albumin**				
≥4.0 g/dL	Reference		Reference	–
<4.0 g/dL	1.41 (1.06 - 1.88)	0.020	1.64 (1.21 - 2.23)	0.001
Not recorded	1.05 (0.79 - 1.41)	0.7	1.18 (0.86 - 1.62)	0.31
**Number of metastatic sites**				
1	Reference	—		
2	1.08 (0.84 - 1.38)	0.4		
3	0.91 (0.65 - 1.28)	0.5		
≥4	0.73 (0.34 - 1.56)	0.6		
**Liver metastasis (no vs. yes)**	1.56 (1.23 - 1.98)	<0.001	1.24 (0.96 - 1.61)	0.09
**Lung metastasis (no vs. yes)**	1.21 (0.94 - 1.55)	0.14		
**Peritoneum metastasis (no vs. yes)**	1.02 (0.80 - 1.31)	0.9		
**Distant lymph node metastasis (no vs. yes)**	1.00 (0.78 - 1.27)	0.9		
**CA19-9**				
< median (921 U/ml)	Reference		Reference	–
≥ median (921 U/ml)	2.20 (1.72 - 2.80)	<0.001	1.79 (1.38 - 2.31)	<0.001
Not recorded	1.39 (0.89 - 2.18)	0.2	1.06 (0.72 – 1.56)	0.78
**Prior exposure of mFOLFIRINOX**	1.69 (1.13 - 2.51)	0.010	0.92 (0.48 - 1.77)	0.81
**Prior exposure of nab-paclitaxel + gemcitabine**	1.00 (0.77 - 1.29)	0.9		
**Prior exposure of SLOG**	1.42 (1.10 - 1.85)	0.007	1.27 (0.90 - 1.81)	0.18
**Prior exposure of gemcitabine + S1**	1.04 (0.81 - 1.32)	0.8		
**Prior exposure of S1 containing regimen**	1.14 (0.90 - 1.45)	0.3		
**Prior exposure of fluorouracil-containing regimen**	1.39 (1.05 - 1.83)	0.021	1.57 (1.12 - 2.20)	0.009
**Prior exposure of irinotecan containing regimen**	1.73 (1.26 - 2.38)	<0.001	1.34 (0.80 - 2.24)	0.26
**Prior exposure of platinum containing regimen**	1.44 (1.16 - 1.80)	0.001	1.21 (0.87 - 1.69)	0.27
**Prior lines of advanced diseases**	1.13 (1.00 - 1.29)	0.059		
**Prior surgery**				
No surgery	Reference		Reference	–
Whipple operation	0.55 (0.39 - 0.76)	<0.001	0.66 (0.46 - 0.95)	0.03
Other surgical procedure	0.69 (0.53 - 0.90)	0.006	0.72 (0.55 - 0.96)	0.02
**Time since last previous therapy**	0.96 (0.87 - 1.06)	0.4		
**Relative 6-week cumulative dose**				
>80%	Reference		Reference	–
60-80%	0.97 (0.73 - 1.29)	0.8	0.94 (0.70 - 1.28)	0.71
<60%	1.89 (1.46 - 2.45)	<0.001	2.12 (1.60 - 2.81)	<0.001

### Dosing Pattern and Cumulative Dose

**Figure 3:**
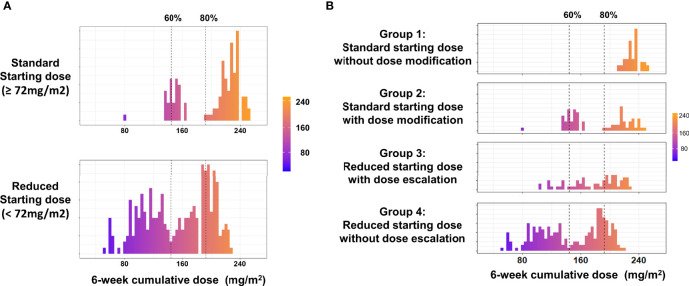
**(A)** Distribution of the 6-week cumulative dose in the standard and reduced starting dose groups. **(B)** Distribution of the 6-week cumulative dose in the different dosing pattern groups. Only included patients received at least two doses of nal-IRI+5FU/LV.

We further analyzed the different dosing patterns to address the possible cause of the varying 6-week cumulative dose. Given that physicians might adjust the dose of nal-IRI by only 10% in real-world practice, we therefore defined a >10% reduction in the starting dose (<72 mg/m^2^) as the reduced starting dose and a >10% decrease or increase in the subsequent cycles as either dose reduction or escalation, respectively. Dose delay was defined as a delay of ≥7 days from the planned dose of every 2 weeks. To reduce bias, patients who received only one dose of nal-IRI+5-FU/LV were excluded from the dosing pattern subgroup analysis as they had no chance for dose modification. Overall, 159 patients started with a standard dose, whereas 274 patients had a reduced starting dose. A bimodal distribution of the 6-week cumulative dose was observed in both the reduced starting dose and standard starting dose groups (**Figure 3A**). Based on the above definition, the patients were further classified into four dosing pattern groups as follows: standard starting dose without dose modification (group 1, n=69), standard starting dose with dose delay or reduction (group 2, n=90), reduced starting dose with dose escalation (group 3, n=63), and reduced starting dose without dose escalation (group 4, n=211). Among 159 patients with a standard starting dose (groups 1 and 2), 90 (56.6%) had early dose modification, which was consistent with that in the nal-IRI + 5-FU/LV arm of the NAPOLI-1 study (53 per 102 patients; 52.0%, p=0.52) (21). Furthermore, there were more patients with stage I–III at diagnosis in groups 1 and 4, whereas more patients in groups 1 and 2 did not have albumin data before nal-IRI+5-FU/LV treatment. Otherwise, the baseline characteristics were not significantly different among the four dosing groups (**Supplementary Table S2**).

**Figure 4:**
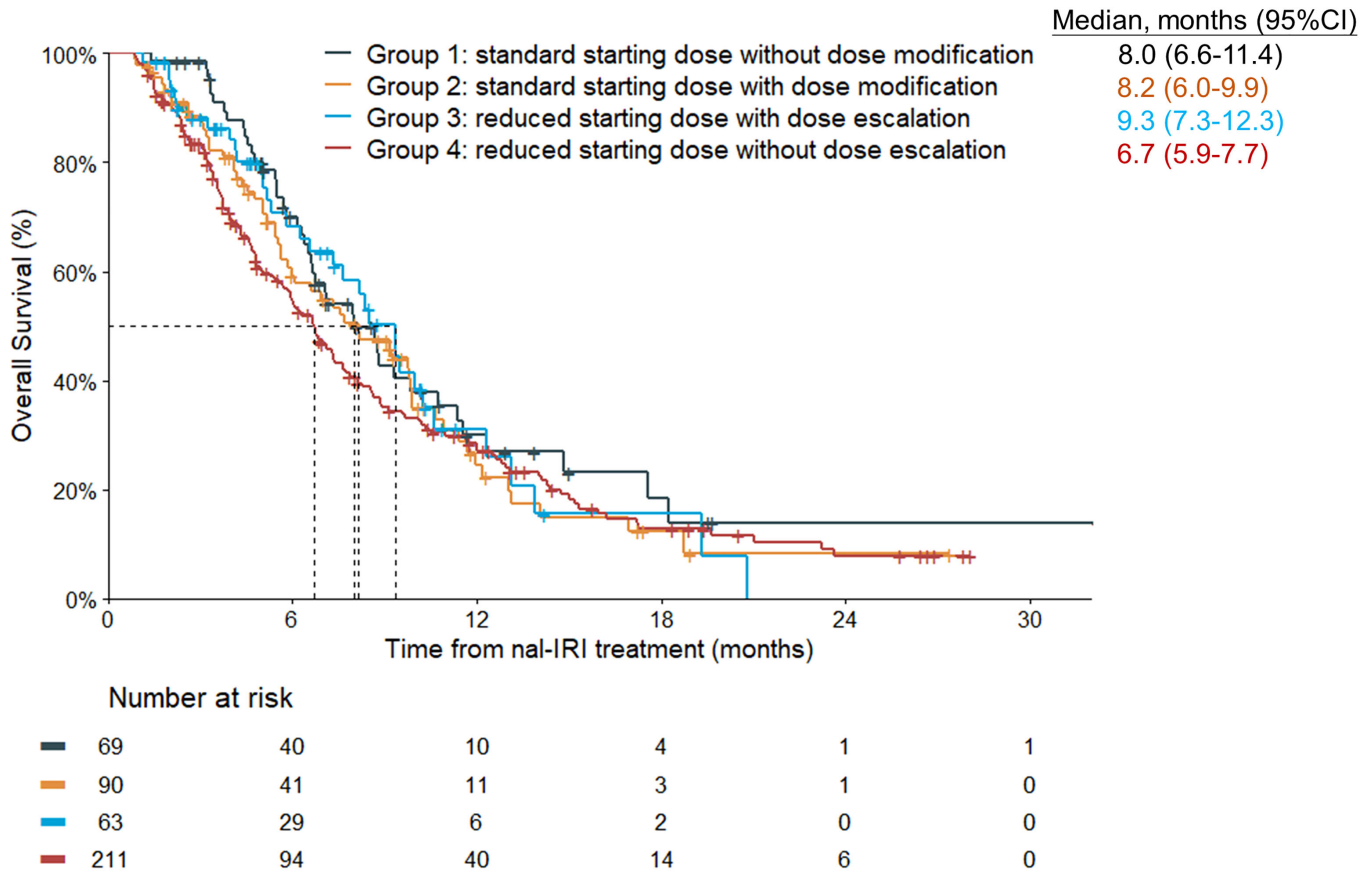
The Kaplan–Meier plot of OS in the different dosing pattern groups. Only included patients received at least two doses of nal-IRI+5FU/LV.

The median (IQR) starting doses in groups 1 and 2 were 79.5 (76.3–80.1) and 79.5 (76.9–80.0) mg/m^2^, respectively; these were 58.5 (48.6–62.9) mg/m^2^ and 60.7 (44.1–65.3) mg/m^2^ in groups 3 and 4 (**Table 4**). The corresponding median 6-week cumulative doses of nal-IRI for dosing pattern groups 1, 2, 3, and 4 were 236, 160, 193, and 153 mg/m^2^, respectively (**Table 4** and **Figure 3B**). Groups 1, 2, and 3 had similar mOS, which was significantly better than that of group 4 (8.0, 8.2, 9.3, and 6.7 months, respectively) (**Figure 4**).

**Table 4:** Dose distribution of different dosing pattern subgroups.

Parameter	6-week cumulative dose, median (IQR), mg/m^2^	Starting dose, median (IQR), mg/m^2^	mOS (95% CI), months
Group 1: standard starting dose without dose modification (N = 69)	236 (230–241)	79.5 (76.3–80.1)	8.0 (6.6–11.4)
Group 2: standard starting dose with dose modification (N = 90)	160 (152–216)	79.5 (76.9–80.0)	8.2 (6.0–9.9)
Group 3: reduced starting dose with dose escalation (N = 63)	193 (157–207)	58.5 (48.6–62.9)	9.3 (7.3–12.3)
Group 4: reduced starting dose without dose escalation (N = 211)	153 (109–187)	60.7 (44.1–65.3)	6.7 (5.9–7.7)

### Real-World Safety Profile

The selected adverse events are listed in **Table 5**. The most common ≥grade 3 adverse events were neutropenia (23.3%), anemia (19.5%), and hypokalemia (12.7%). All adverse events were comparable to those observed in the NAPOLI-1 study without extra safety considerations in real-world settings except for more ≥grade 3 anemia (19.5% vs. 9.4%) and less ≥grade 3 diarrhea in our study (2.7% vs. 12.8%). There was no significant difference in the safety profile among the three cumulative dose groups.

**Table 5:** Adverse effects in different 6-week cumulative dose groups.

	NAPOLI-1 Nal-IRI+5-FU/LV (N = 117)	Real-world cohort			
		Overall (N = 473)	Relative 6-week cumulative dose >80% (N = 175)	Relative 6-week cumulative dose 60-80% (N = 134)	Relative 6-week cumulative dose <60% (N = 164)
**Neutropenia**					
All grade	46 (39.3%)	197 (41.6%)	88 (50.3%)	53 (39.6%)	56 (34.1%)
≥Grade 3	32 (27.4%)	110 (23.3%)	51 (29.1%)	29 (21.6%)	30 (18.3%)
Febrile neutropenia	2 (1.71%)	16 (3.4%)	7 (4.0%)	7 (5.2%)	2 (1.2%)
Not recorded	-	9 (1.9%)	0 (0%)	2 (1.5%)	7 (4.3%)
**Anemia**					
All grades	44 (37.6%)	301 (63.6%)	118 (67.4%)	92 (68.7%)	91 (55.5%)
≥Grade 3	11 (9.4%)	92 (19.5%)	36 (20.6%)	26 (19.4%)	30 (18.3%)
Not recorded	-	6 (1.3%)	1 (0.6%)	1 (0.7%)	4 (2.4%)
**Hypokalemia**					
All grades	14 (12.0%)	152 (32.1%)	60 (34.3%)	45 (33.6%)	47 (28.7%)
≥Grade 3	4 (3.4%)	60 (12.7%)	22 (12.6%)	15 (11.2%)	23 (14.0%)
Not recorded	-	105 (22.2%)	46 (26.3%)	29 (21.6%)	30 (18.3%)
**Fatigue**					
All grades	47 (40.2%)	211 (44.6%)	89 (50.9%)	59 (44.0%)	63 (38.4%)
≥Grade 3	16 (13.7%)	8 (1.7%)	1 (0.6%)	3 (2.2%)	4 (2.4%)
Not recorded	-	35 (7.4%)	12 (6.9%)	8 (6.0%)	15 (9.1%)
**Vomiting**					
All grades	61 (52.1%)	186 (39.3%)	74 (42.3%)	60 (44.8%)	52 (31.7%)
≥Grade 3	13 (11.1%)	15 (3.2%)	2 (1.1%)	4 (3.0%)	9 (5.5%)
Not recorded	-	9 (1.9%)	2 (1.1%)	2 (1.5%)	5 (3.0%)
**Diarrhea**					
All grades	69 (59.0%)	142 (30.0%)	54 (30.9%)	49 (36.6%)	39 (23.8%)
≥Grade 3	15 (12.8%)	13 (2.7%)	2 (1.1%)	4 (3.0%)	7 (4.3%)
Not recorded	-	13 (2.7%)	4 (2.3%)	3 (2.2%)	6 (3.7%)

Of note, despite the 6-week cumulative dose in dosing pattern group 3 being numerically higher than that in group 2, the former had significantly less all-grade neutropenia (34.9% vs. 58.9%, p=0.005) and less ≥grade 3 neutropenia (15.9% vs. 34.4%, p=0.015) than the latter (**Supplementary Table S3** **).**

## Discussion

Through the collaboration of nine medical centers in Taiwan, we collected one of the largest numbers of patients with previous gemcitabine-refractory mPDAC who had received nal-IRI+5-FU/LV treatment. With similar patient demographics, the treatment response and survival in our study were comparable to those in the NAPOLI-1 study. Thus, our study provides real-world evidence to support the effectiveness and safety of nal-IRI+5-FU/LV in gemcitabine-refractory mPDAC.

In the pre-specified analysis of the NAPOLI-1 study, the PP population in the nal-IRI+5-FU/LV arm, defined as ≥80% of the planned treatment during the first 6 weeks, had an mOS of 8.9 months; it was significantly better than that in the non-PP population (4.4 months) (20). In this study, the corresponding mOS of patients with >80% and ≤80% of the planned nal-IRI dose within 6 weeks were 7.9 and 6.3 months, respectively (p=0.0071). The mOS of patients with relative 6-week cumulative doses of 60%–80% and <60% were 8.2 and 4.3 months, respectively. Although our study set a different cut-off point at 60% as compared to the 80% proposed in the NAPOLI-1 PP study, our results were still consistent with the NAPOLI-1 PP study that achieving a certain level of cumulative nal-IRI dose within first 6 weeks, an indicator of treatment compliance to nal-IRI+5-FU/LV treatment, could impact their survival outcome.

It is hypothesized that the longer survival of patients with a higher 6-week cumulative dose was due to better baseline conditions. For example, in the nal-IRI+5-FU/LV arm of the NAPOLI-1 study, the percentage of patients with a Karnofsky PS of 90–100 in the PP and non-PP populations were 62.1% and 49%, respectively (20). However, in our study, the impact of the 6-week cumulative dose of nal-IRI on survival remained significant even after adjusting for confounding factors by Cox regression (**Table 3**). Therefore, these findings justify the importance of dose delivery, regardless of baseline conditions.

In the NAPOLI-1 trial, the starting dose of nal-IRI in the combination arm was fixed at 80 mg/m^2^, except for those with known homozygous UGT1A1*28 alleles. In real-world practice, the starting dose of nal-IRI would likely be modified upon the discretion of the physicians. Furthermore, four of the 11 RWDs reported that pre-emptive starting dose reduction had no impact on survival, while three studies reported a subsequent dose delay or modification that did not jeopardize the treatment outcome (**Supplementary Table S1**) (9, 10, 14–16). Only one study reported that patients with subsequent dose delays or modifications had better survival (15). However, the results should be interpreted with caution since it was a database-derived study and the immortal time bias was not adjusted (15). In our dosing pattern subgroup analysis, the mOS was similar in patients who received the standard starting dose with or without dose modification, with mOS of 8.0 and 8.2 months, respectively. This result is consistent with the findings of the previous *post-hoc* analysis from the NAPOLI-1 study; patients with early dose modification had comparable survival as compared to those without dose modification (mOS of 8.4 vs. 6.7 months, respectively, p=0.595) (21). Furthermore, the reduced starting dose followed by dose escalation in well-tolerated patients is a feasible strategy to maintain adequate dose delivery (median 6-week cumulative dose: 193 mg/m^2^, 80% of the planned dose) and therapeutic outcomes (mOS: 9.3 months), as compared to those receiving standard starting doses with and without early dose modification. The effectiveness of the dose escalation strategy has been demonstrated in oncology. For example, in the ReDOS colon cancer study, a reduced regorafenib starting dose from 80 mg/day with escalation had comparable survival but less toxicity than the standard dose group, which started at 160 mg per day (24). Thus, our study once again highlights the importance of a dose modification strategy to help patients receive adequate dose delivery. Baseline characteristics were not significantly different among the four dosing pattern groups (**Supplementary Table S2**). Physician preference, patient adherence, or a decline in performance could possibly contribute to different dosing patterns; these factors were not recorded in the EMR. However, based on our observation, a lower starting dose followed by a re-escalation strategy could achieve clinical outcomes comparable to those with standard starting doses in real-world practice. The nuance approach should be beneficial in a group of relatively fragile patients with disease progression and possible residual toxicities from previous chemotherapy (25).

This study has some limitations owing to its retrospective nature. First, there is no pre-specified dose modification protocol; we define a 10% dose modification value based on clinical experience and previous real-world studies (13). Second, adverse events in the first 6 weeks could influence patient adherence as well as the 6-week cumulative dose. However, our study recorded adverse events during the entire treatment course without a specified time frame for reporting adverse events in the first 6 weeks. Moreover, the cause of the reduced starting dose was not recorded in the EMRs. This was not only a limitation of our study but also a reflection of real-world practice. Our study suggested that inadequate dose delivery (<60%), regardless of cause, could negatively impact patient survival. Finally, although we adjusted for a variety of possible confounding factors that could influence the dosing pattern, some factors cannot be adjusted owing to its retrospective nature, such as physician preference or patient adherence. Therefore, the success of the dose-escalation strategy observed in our study requires further validation in a prospective randomized controlled study.

## Conclusions

We report one of the largest cohorts of patients with mPDAC who were treated with nal-IRI+5-FU/LV. The treatment outcomes and safety profiles in our study were comparable to those of the NAPOLI-1 study, which supported nal-IRI+5-FU/LV as the standard of care after the failure of gemcitabine-based treatment. Our study also demonstrated the effect of the 6-week cumulative dose on overall survival, thus highlighting the importance of achieving adequate dose delivery. Moreover, our study provided real-world evidence to support a lower starting dose followed by a re-escalation strategy, which can be associated with a lower incidence of significant adverse events as well as comparable survival as compared to starting with a standard dose.

## Data Availability Statement

The raw data supporting the conclusions of this article will be made available by the authors, without undue reservation.

## Ethics Statement

The studies involving human participants were reviewed and approved by Chang Gung Memorial Hospital; China Medical University Hospital; Chung Shan Medical University Hospital; National Cheng Kung University Hospital; National Taiwan University Hospital; Kaohsiung Medical University Hospital; Taipei Veterans General Hospital; Tri-Service General Hospital. Written informed consent for participation was not required for this study in accordance with the national legislation and the institutional requirements.

## Author Contributions

Y-SS and S-CChiu conceived the original idea and design of the work; C-PL, C-JY, S-HY, W-CC, J-SC, T-JC, Y-YC, S-CChuang, L-YB, C-FC, C-MP, D-CH, and Y-HY contributed to the acquisition, analysis, and interpretation of data. Y-YS and N-JC drafted the manuscript. Y-SS and L-TC substantively revised the manuscript. All authors have read and approved the final manuscript.

## Conflict of Interest

Author Sz-CC was employed by PharmaEngine Inc. The article processing charge is kindly sponsored by PharmaEngine Inc.

The remaining authors declare that the research was conducted in the absence of any commercial or financial relationships that could be construed as potential conflicts of interest.

## Publisher’s Note

All claims expressed in this article are solely those of the authors and do not necessarily represent those of their affiliated organizations, or those of the publisher, the editors and the reviewers. Any product that may be evaluated in this article, or claim that may be made by its manufacturer, is not guaranteed or endorsed by the publisher.
