# Supplementary material for: Dosing Pattern and Early Cumulative Dose of Liposomal Irinotecan in Metastatic Pancreatic Cancer: A Real-World Multicenter Study
Source: Front Oncol. 2022 Jun 22;12:800842. doi: 10.3389/fonc.2022.800842 (PMC9256928; doi:10.3389/fonc.2022.800842)
Supplement: Supplementary file 1 [file DataSheet_1.docx]

Supplementary Material

## Supplementary Tables

**Table S1** Summary of real-world evidence of nal-IRI+5-FU/LV in pancreatic cancer

| Reference | Case number | Proportion of ECOG ≥2, n (%) | mPFS (month) | mOS  (month) | ORR | Reduced starting dose | |  | Dose delay or modification | |
| --- | --- | --- | --- | --- | --- | --- | --- | --- | --- | --- |
|  |  |  |  |  |  | n (%) | Survival |  | n (%) | Survival |
| [9] | 56 | 12 (21.4) | 2.9 | 5.3 | 5.4% | 39 (69.6) | No impact |  | 18 (32.1) | No impact |
| [10] | 52 | 1 (1.9) | 3.8 | 6.8 | 19.2% | 1 (1.9) | NA |  | 14 (26.9) | No impact |
| [11] | 86 | 0 | 3.5 | 9.4 | 10.4% | NA | NA |  | 43 (50.0) | NA |
| [12] | 35 | 6 (17.1) | 4.1 | 7.1 | NA | 4 (11.4) | NA |  | 24 (68.6) | NA |
| [13] | 257 | 38 (14.8) | 2.3† | 4.9 | NA | 67 (26.1) | NA |  | 70 (27.2) | NA |
| [14] | 44 | 12 (27.3) | 2.5 | 6.6 | 9.1% | 29 (65.9) | No impact |  | 9 (20.5) | NA |
| [15] | 320 | 54 (16.9) | 1.9† | 4.3 | NA | 128 (36.8) ‡ | No impact |  | 91 (28.4) | Better¶ |
| [16] | 58 | 4 (6.9) | 2.7 | 5.4 | 10.3% | 15 (25.9) | No impact |  | 24 (41.4) | No impact |
| [17] | 104 | 18 (17.3) | 3.7 | 7.7 | 11.5% | 35 (33.7) | NA |  | NA | NA |
| [18]§ | 35 | 7 (20.0) | 2.0 | 4.4 | 2.9% | NA | NA |  | NA | NA |
| [19] | 374 | 81 (21.7) | 2.5 | 6.1 | NA | NA | NA |  | 108 (29.4) | NA |
| ECOG: Eastern Cooperative Oncology Group performance score; mPFS: median progression free survival; mOS: median overall survival; ORR: objective response rate; NA: not available  † Time to treatment discontinuation.  ‡ Only reported in the 348 patients validation cohort but not in the main cohort.  § All patients had received non-liposomal irinotecan prior to nal-IRI.  ¶ Patients with dose delay or modification had better overall survival with a hazard ratio of 0.67 (95% Cl: 0.49–0.91). | | | | | | | | | | |

**Table S2. Baseline characteristics of different dosing pattern groups (N=433)**

|  | **Group 1 (N=69)**  standard starting dose without dose modification | **Group 2 (N=90)**  standard starting dose  with dose modification | **Group 3 (N=63)**  reduced starting dose  with dose escalation | **Group 4 (N=211)**  reduced starting dose  without dose escalation |
| --- | --- | --- | --- | --- |
| **Gender** |  |  |  |  |
| Female | 35 (50.7%) | 39 (43.3%) | 22 (34.9%) | 85 (40.3%) |
| Male | 34 (49.3%) | 51 (56.7%) | 41 (65.1%) | 126 (59.7%) |
| **Age**, year, median (range) | 62.0 (42.0 - 80.0) | 63.0 (33.0 - 81.0) | 64.0 (44.0 - 81.0) | 62.0 (27.0 - 86.0) |
| **Disease stage at diagnosis** |  |  |  |  |
| Stage I-III | 26 (37.7%) | 19 (21.1%) | 13 (20.6%) | 85 (40.3%) |
| Stage IV | 43 (62.3%) | 71 (78.9%) | 50 (79.4%) | 126 (59.7%) |
| **Primary tumor location**† |  |  |  |  |
| Head | 31 (44.9%) | 51 (56.7%) | 35 (55.6%) | 111 (52.6%) |
| Body | 21 (30.4%) | 12 (13.3%) | 12 (19.0%) | 54 (25.6%) |
| Tail | 15 (21.7%) | 25 (27.8%) | 15 (23.8%) | 30 (14.2%) |
| Multi-locations including head | 2 (2.9%) | 2 (2.2%) | 1 (1.6%) | 13 (6.2%) |
| Multi-locations excluding head | 0 (0%) | 0 (0%) | 0 (0%) | 3 (1.4%) |
| **Baseline albumin** |  |  |  |  |
| <4.0 g/dL | 23 (33.3%) | 22 (24.4%) | 22 (34.9%) | 96 (45.5%) |
| ≥4.0 g/dL | 9 (13.0%) | 18 (20.0%) | 26 (41.3%) | 52 (24.6%) |
| Not recorded | 37 (53.6%) | 50 (55.6%) | 15 (23.8%) | 63 (29.9%) |
| **Number of metastatic sites** |  |  |  |  |
| 1 | 32 (46.4%) | 49 (54.4%) | 35 (55.6%) | 121 (57.3%) |
| 2 | 21 (30.4%) | 27 (30.0%) | 17 (27.0%) | 63 (29.9%) |
| 3 | 14 (20.3%) | 12 (13.3%) | 9 (14.3%) | 20 (9.5%) |
| ≥4 | 2 (2.9%) | 2 (2.2%) | 2 (3.2%) | 7 (3.3%) |
| **Sites of metastases** |  |  |  |  |
| Liver | 42 (60.9%) | 54 (60.0%) | 47 (74.6%) | 144 (68.2%) |
| Lung | 21 (30.4%) | 23 (25.6%) | 16 (25.4%) | 49 (23.2%) |
| Peritoneum | 17 (24.6%) | 23 (25.6%) | 18 (28.6%) | 61 (28.9%) |
| Distant lymph node | 28 (40.6%) | 28 (31.1%) | 13 (20.6%) | 55 (26.1%) |
| **CA 19-9** |  |  |  |  |
| <40 U/ml | 9 (13.0%) | 17 (18.9%) | 8 (12.7%) | 30 (14.2%) |
| ≥40 U/ml | 52 (75.4%) | 61 (67.8%) | 47 (74.6%) | 155 (73.5%) |
| Not recorded | 8 (11.6%) | 12 (13.3%) | 8 (12.7%) | 26 (12.3%) |
| **Previous anticancer therapy** |  |  |  |  |
| mFOLFIRINOX | 9 (5.8%) | 16 (9.6%) | 2 (5.7%) | 11 (9.5%) |
| Gemcitabine + nab-paclitaxel | 15 (21.7%) | 19 (21.1%) | 28 (44.4%) | 72 (34.1%) |
| SLOG | 43 (27.7%) | 40 (24.0%) | 4 (11.4%) | 10 (8.6%) |
| Gemcitabine + S1 | 50 (32.3%) | 47 (28.1%) | 9 (25.7%) | 26 (22.4%) |
| Gemcitabine-containing | 155 (100%) | 167 (100%) | 35 (100%) | 116 (100%) |
| Fluorouracil-containing | 127 (81.9%) | 136 (81.4%) | 28 (80.0%) | 75 (64.7%) |
| S1-containing | 115 (74.2%) | 121 (72.5%) | 21 (60.0%) | 60 (51.7%) |
| Irinotecan-containing | 13 (8.4%) | 21 (12.6%) | 9 (25.7%) | 21 (18.1%) |
| Platinum-containing | 77 (49.7%) | 92 (55.1%) | 12 (34.3%) | 37 (31.9%) |
| **Prior lines of advanced diseases** ‡ |  |  |  |  |
| 0 | 0 (0%) | 1 (1.1%) | 2 (3.2%) | 2 (0.9%) |
| 1 | 49 (71.0%) | 68 (75.6%) | 33 (52.4%) | 119 (56.4%) |
| ≥2 | 20 (29.0%) | 21 (23.3%) | 28 (44.4%) | 90 (42.7%) |
| **Prior surgery** |  |  |  |  |
| No surgery | 39 (56.5%) | 56 (62.2%) | 40 (63.5%) | 121 (57.3%) |
| Whipple operation | 12 (17.4%) | 20 (22.2%) | 12 (19.0%) | 28 (13.3%) |
| Other surgical procedure | 18 (26.1%) | 14 (15.6%) | 11 (17.5%) | 62 (29.4%) |
| **Time since last previous therapy** |  |  |  |  |
| Median (IQR), months | 0.689 (0.459 - 1.31) | 0.689 (0.557 - 1.02) | 0.525 (0.426 - 0.885) | 0.689 (0.459 - 1.15) |
| Not recorded | 4 (5.8%) | 9 (10.0%) | 20 (31.7%) | 28 (13.3%) |
| IQR: interquartile range; NA: not available.  †In NAPOLI-1 study, 2.7% of patients are classified as unknown location which is not shown here.  ‡The definition is slightly different. Systemic therapy used for locally advanced disease is counted in the present real-world study, but not in NAPOLI-1 study. | | | | |

**Table S3** Adverse effects in different dosing strategy group

|  | **Group 1 (N=69)**  standard starting dose without dose modification | **Group 2 (N=90)**  standard starting dose  with dose modification | **Group 3 (N=63)**  reduced starting dose  with dose escalation | **Group 4 (N=211)**  reduced starting dose  without dose escalation |
| --- | --- | --- | --- | --- |
| **Neutropenia** |  |  |  |  |
| All grade | 35 (50.7%) | 53 (58.9%) | 22 (34.9%) | 81 (38.4%) |
| ≥ grade 3 | 22 (31.9%) | 31 (34.4%) | 10 (15.9%) | 43 (20.4%) |
| Febile neutropenia | 2 (2.9%) | 6 (6.7%) | 2 (3.2%) | 6 (2.8%) |
| Not recorded | 0 (0%) | 1 (1.1%) | 0 (0%) | 3 (1.4%) |
| **Anemia** |  |  |  |  |
| All grade | 41 (59.4%) | 61 (67.8%) | 45 (71.4%) | 138 (65.4%) |
| ≥ grade 3 | 18 (26.1%) | 17 (18.9%) | 11 (17.5%) | 44 (20.9%) |
| Not recorded | 1 (1.4%) | 1 (1.1%) | 0 (0%) | 0 (0%) |
| **Thrombocytopenia** |  |  |  |  |
| All grade | 17 (24.6%) | 25 (27.8%) | 18 (28.6%) | 52 (24.6%) |
| ≥ grade 3 | 2 (2.9%) | 4 (4.4%) | 2 (3.2%) | 15 (7.1%) |
| Not recorded | 0 (0%) | 1 (1.1%) | 0 (0%) | 1 (0.5%) |
| **AST or ALT increased** |  |  |  |  |
| All grade | 19 (27.5%) | 36 (40.0%) | 23 (36.5%) | 57 (27.0%) |
| ≥ grade 3 | 2 (2.9%) | 6 (6.7%) | 1 (1.6%) | 3 (1.4%) |
| Not recorded | 20 (29.0%) | 20 (22.2%) | 18 (28.6%) | 44 (20.9%) |
| **Blood bilirubin increased** |  |  |  |  |
| All grade | 9 (13.0%) | 24 (26.7%) | 15 (23.8%) | 46 (21.8%) |
| ≥ grade 3 | 4 (5.8%) | 10 (11.1%) | 7 (11.1%) | 12 (5.7%) |
| Not recorded | 7 (10.1%) | 8 (8.9%) | 2 (3.2%) | 17 (8.1%) |
| **Creatinine increased** |  |  |  |  |
| All grade | 15 (21.7%) | 12 (13.3%) | 8 (12.7%) | 39 (18.5%) |
| ≥ grade 3 | 0 (0%) | 0 (0%) | 0 (0%) | 3 (1.4%) |
| Not recorded | 4 (5.8%) | 5 (5.6%) | 3 (4.8%) | 9 (4.3%) |
| **Hypokalemia** |  |  |  |  |
| All grade | 25 (36.2%) | 37 (41.1%) | 17 (27.0%) | 64 (30.3%) |
| ≥ grade 3 | 8 (11.6%) | 14 (15.6%) | 5 (7.9%) | 30 (14.2%) |
| Not recorded | 12 (17.4%) | 19 (21.1%) | 18 (28.6%) | 47 (22.3%) |
| **Fatigue** |  |  |  |  |
| All grade | 38 (55.1%) | 44 (48.9%) | 27 (42.9%) | 89 (42.2%) |
| ≥ grade 3 | 0 (0%) | 2 (2.2%) | 1 (1.6%) | 2 (0.9%) |
| Not recorded | 5 (7.2%) | 16 (17.8%) | 1 (1.6%) | 3 (1.4%) |
| **Vomiting** |  |  |  |  |
| All grade | 32 (46.4%) | 47 (52.2%) | 20 (31.7%) | 76 (36.0%) |
| ≥ grade 3 | 0 (0%) | 2 (2.2%) | 0 (0%) | 12 (5.7%) |
| Not recorded | 1 (1.4%) | 3 (3.3%) | 0 (0%) | 0 (0%) |
| **Diarrhea** |  |  |  |  |
| All grade | 32 (46.4%) | 47 (52.2%) | 20 (31.7%) | 76 (36.0%) |
| ≥ grade 3 | 0 (0%) | 2 (2.2%) | 1 (1.6%) | 10 (4.7%) |
| Not recorded | 1 (1.4%) | 4 (4.4%) | 1 (1.6%) | 2 (0.9%) |
| **Hypoalbuminemia** |  |  |  |  |
| All grade | 15 (21.7%) | 21 (23.3%) | 19 (30.2%) | 60 (28.4%) |
| ≥ grade 3 | 1 (1.4%) | 2 (2.2%) | 0 (0%) | 3 (1.4%) |
| Not recorded | 7 (10.1%) | 12 (13.3%) | 8 (12.7%) | 31 (14.7%) |

## Supplementary Figures


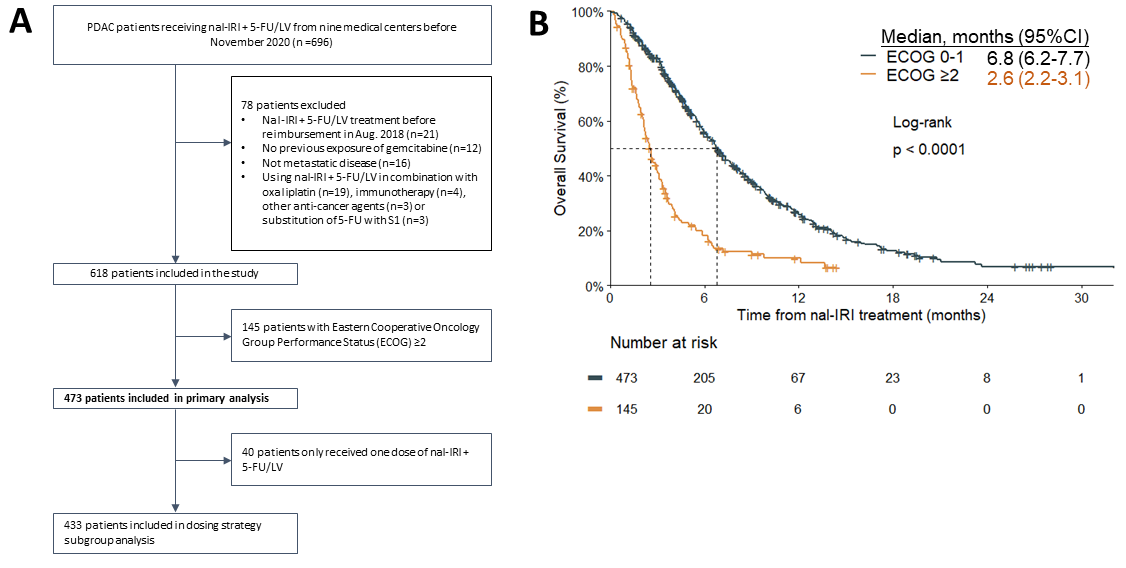


**Supplementary Figure S1.** Overall survival in patients with different performance status.
